# A generic intelligent tomato classification system for practical applications using DenseNet-201 with transfer learning

**DOI:** 10.1038/s41598-021-95218-w

**Published:** 2021-08-04

**Authors:** Tao Lu, Baokun Han, Lipin Chen, Fanqianhui Yu, Changhu Xue

**Affiliations:** 1 College of Mechanical and Electronic Engineering, Shandong University of Science and Technology, Qingdao, 266590 China; 2 School of Mechanical and Automotive Engineering, Qingdao University of Technology, Qingdao, 266520 China; 3 College of Food Science and Engineering, Ocean University of China, Qingdao, 266003 China; 4 Laboratory of Marine Drugs and Biological Products, Pilot National Laboratory for Marine Science and Technology (Qingdao), Qingdao, 266237 China

**Keywords:** Plant sciences, Engineering

## Abstract

A generic intelligent tomato classification system based on DenseNet-201 with transfer learning was proposed and the augmented training sets obtained by data augmentation methods were employed to train the model. The trained model achieved high classification accuracy on the images of different quality, even those containing high levels of noise. Also, the trained model could accurately and efficiently identify and classify a single tomato image with only 29 ms, indicating that the proposed model has great potential value in real-world applications. The feature visualization of the trained models shows their understanding of tomato images, i.e., the learned common and high-level features. The strongest activations of the trained models show that the correct or incorrect target recognition areas by a model during the classification process will affect its final classification accuracy. Based on this, the results obtained in this study could provide guidance and new ideas to improve the development of intelligent agriculture.

## Introduction

Deep learning, an emerging non-destructive technique with advantages of automation, speed, accuracy and low cost, has been successfully applied in agriculture and food fields such as pest detection, disease diagnosis, ripeness determination and quality assessment of fruits, vegetables, meat and seafood^1–3^. In particular, deep convolutional neural networks (CNN) have become the dominant deep learning approach in image-based recognition, classification, and detection tasks for fruits and vegetables^4–6^. With its outstanding ability of automatically learning features from images, CNN-based approaches have been integrated with existing agricultural practices to accelerate the development of smart farming and precision agriculture^7^. Specifically, applying CNN to harvesting robots can guide them to detect and distinguish different types of fruits in an orchard, such as different varieties, maturity stages or grades of fruits, which helps robot to pick accurately and quickly, and reduce labor costs^8–10^. In recent years, a number of studies have been devoted to investigating the feasibility and applicability of CNN in fruit image-based detection and classification. However, many studies have ignore the differences between laboratory use and real-world use of deep learning because they only use the ideal images captured in laboratory environments, i.e., high-resolution images with a white background taken by a well-set-up digital camera, which do not reproduce the range of conditions in practice^11,12^. Although a few studies have used images that closely relate to reality, such as fruits with leaves or a bunch of fruit, they still miss the most important point in practice, which is unforeseen circumstances^13^.

In general, CNN is trained and tested on high quality image datasets, but in practice, it cannot be assumed that the input images are all high quality^14^. This is because in practical environments, image noise is inevitable due to the various processes involved in image acquisition, conversion and transmission^15^. Image noise is a number of isolated, randomly positioned pixels that do not reflect the true information of an image^16^. It is the major contributor to poor image quality and loss of useful information and signals, usually caused by photography equipment and the external environment^17^. For example, unclear images are taken by digital cameras with inherent noise, dirty lenses, or working in fog, rain or snow. And blurry images are taken by robotic vision system because the fruit is obscured by leaves or is blown by the wind^18^. As a result, poor quality images with different levels of noise are often collected in agricultural production. Unfortunately, the obtained poor quality images can strongly interfere with CNN’s target detection and classification, degrading its performance, and leading to inaccurate predictions of the output^14,19^. Based on this, the real-world applications of CNN are more challenging, as it requires CNN-based methods to be more generalizable and robust.

Inspired and motivated by the above reasons, the purpose of this study was to address the problem of low accuracy of CNN in identifying and classifying poor quality images in practical applications. For this reason, DenseNet-201 with transfer learning was employed to develop models, and data augmentation methods were used to enhance and expand the size of training sets. This study contributes to the further advancement of CNN-based methods from laboratory applications to actual agricultural production processes, such as the establishment of CNN-based automated systems for fruit and vegetable picking, sorting and packing, which will facilitate the development of intelligent agriculture in the future and improve labor efficiency and economic benefits.

## Material and methods

### Fruits-360 dataset

“Fruits-360” (https://www.kaggle.com/moltean/fruits, Version: 2020.05.18.0) is a large and open benchmark fruit images dataset^20,21^, which has been employed by several studies to evaluate their proposed models^22,23^. Based on this, “Fruits-360” dataset was employed in this study to objectively evaluate and demonstrate the performance of our proposed models and to facilitate researchers to reproduce our work. This dataset contains a total number of 90,483 images of fruits and vegetables in 131 classes. Among them, tomato includes 9 types, namely Tomato 1, Tomato 2, Tomato 3, Tomato 4, Cherry Red, Heart, Maroon, Tomato not Ripened, and Yellow. Each image (100 × 100 pixels) is of a single tomato on a white background.

### DenseNet architecture

DenseNet was proposed by Huang et al.^24^, and is known for its excellent performance on four object recognition benchmark datasets such as CIFAR-100 and ImageNet^25^. To maximize the information flow between the layers in the network, the DenseNet architecture uses a simple connectivity pattern that connects all layers directly to each other in a feed-forward fashion, i.e., each layer obtains additional inputs from all previous layers and passes its own feature-maps to all subsequent layers^26^. With this architecture, DenseNet has several impressive advantages, including mitigating the vanishing gradient problem, strengthening feature propagation, encouraging feature reuse, and substantially reducing the number of parameters. As a result, DenseNet-201 was employed in this study and more details can be found in^24^.

### Transfer learning

CNN is typically exploited on large datasets of more than one million images (e.g., ImageNet) and perform best when they have deeper and more highly interconnected layers^27^. However, it is difficult to obtain a huge number of manually labeled images in agriculture, so the currently used CNN-based methods for agricultural issues such as fruit and vegetable classification are directly exploited on a limited number of classes and small datasets, which can easily lead to overfitting problems of deep networks, and thus the results obtained are not rigorous and scientific^28^. An effective way to overcome overfitting problems while achieving significant results in classification tasks with a limited amount of data is transfer learning^26^. Transfer learning is a deep learning approach in which a model that trained for one task is used as a starting point to train a model for a second task. With its help, deep CNN can not only avoid overfitting problems when the dataset is relatively small, but also reduce training time.

### Image processing

As the original size of each image in Fruits-360 dataset was 100 × 100 × 3, all images were resized to 224 × 224 × 3 to comply with the input size requirements of DenseNet-201.

### Data augmentation by adding Gaussian white noise to images

Data augmentation is a popular technique used to enhance the training of CNN^29^. Data augmentation mitigates the overfitting problems of deep networks on small datasets because it expands the size of the dataset. The commonly used data augmentation methods include geometric rotation, adversarial training, and generative adversarial networks, etc^30^. However, these methods also have some problems, such as geometrically rotated images cannot solve the problem of low accuracy of CNN in identifying images with noise, while generative adversarial networks are relatively complex and hard to train^31^. Additive Gaussian white noise is a fundamental noise model used in *Information Theory* to mimic the effect of many random processes that occur in nature^32^. In addition, injecting Gaussian white noise into images can be used as a simple and convenient way to augment the dataset. Therefore, in order to expand the size of dataset and simulate different levels of poor-quality images acquired in practical scenarios, data augmentation was performed by adding Gaussian white noise with mean *M* and variance of 0.01, where *M* ranges from 0 to 1.0.

### Feature visualization and strongest activations

Feature visualization images of the last fully connected layer of each trained model were generated by the *deepDreamImage* technique^33^. Strongest activations images of the last convolutional layer of each trained model were generated using the method “*Visualize Activations of a Convolutional Neural Network*” in Mathworks (R2020b).

### Computer configuration and operating parameters

All models were implemented using the MATLAB R2020b version, ran on the same workstation with Intel Xeon Gold 5120 CPU*2, Nvidia P2000 GPU (5 G memory) *1, and 64 G (16 G*4) memory. Models were trained by Adaptive Moment Estimation (ADAM). In addition, the same operating parameters were adopted: initial learn rate = 0.00001, minibatch size = 64, and max epochs = 2.

## Results and discussion

### Performance comparision of five CNN-based models

In order to select the optimal model, the performance of the five CNN-based models (NasNet-Mobile, Xception, DenseNet-201, Inception-Resnetv2, and Inception-v3 with transfer learning) was evaluated on different datasets (Tables 1, 2), and the results are presented in Table 3. Specifically, the five models were first trained and tested on the original dataset (training set 1—testing set 1) provided by “Fruits-360”, respectively, and all models achieved high classification accuracy of around 99%, especially Xception, DenseNet-201, and Inception-Resnetv2-based models achieved almost 100% classification accuracy with no significant difference. Therefore, for further comparison, the five models were then trained and tested on training set 2—testing set 2, which is an inverse version of training set 1—testing set 1, i.e., training set 2 is testing set 1, and testing set 2 is training set 1. This kind of dataset configuration posed a challenge for the five models since the size of training set was reduced and the size of testing set was increased (Table 1). Undoubtedly, the results showed a significant decrease in classification accuracy for most models, but DenseNet-201-based model still achieved the best performance with the highest classification accuracy of 96.16%. Based on this, DenseNet-201-based model was employed in this study for further discussion.

**Table 1:** The number of images used in different training sets.

Tomato type	Training set 1	Training set 2	Training set 3	Training set 4	Training set 5
Tomato 1	738	246	1476	2952	2952
Tomato 2	672	225	1344	2688	2688
Tomato 3	738	246	1476	2952	2952
Tomato 4	479	160	958	1916	1916
Cherry red	492	164	984	1968	1968
Heart	684	228	1368	2736	2736
Maroon	367	127	734	1468	1468
Tomato not ripened	474	158	948	1896	1896
Yellow	459	153	918	1836	1836
Total	5103	1707	10,206	20,412	20,412

For each type of tomato in each training set, 1/5 of the images were used for validation and the remaining 4/5 of the images were used for training.

**Table 2:** The number of images used in different testing sets.

Tomato type	Testing set 1	Testing set 2	Testing set 3 (*M* = 0)	…	Testing set 13 (*M* = 1.0)
Tomato 1	246	738	246	…	246
Tomato 2	225	672	225	…	225
Tomato 3	246	738	246	…	246
Tomato 4	160	479	160	…	160
Cherry red	164	492	164	…	164
Heart	228	684	228	…	228
Maroon	127	367	127	…	127
Tomato not ripened	158	474	158	…	158
Yellow	153	459	153	…	153
Total	1707	5103	1707	…	1707

A total of 11 testing sets, from testing set 3 to 13, were added with different levels of noise, and the noise addition was increased from *M* = 0 to *M* = 1.0, with an increment of 0.1.

**Table 3:** Performance comparison of five CNN-based models on different datasets.

	NasNet-Mobile	Xception	DenseNet-201	Inception-Resnetv2	Inception-v3
**Training set 1: Testing set 1**					
Accuracy (%)	98.95	99.94	100.00	99.94	99.12
Training time (s)	24,875	49,007	28,706	55,022	12,030
Testing time (s)	126	119	53	121	29
**Training set 2: Testing set 2**					
Accuracy (%)	88.97	91.63	96.16	90.40	92.16
Training time (s)	8126	17,704	13,340	21,347	4397
Testing time (s)	478	876	157	392	80

### Influence of image noise on performance of DenseNet-201-based model

**Figure 1:**
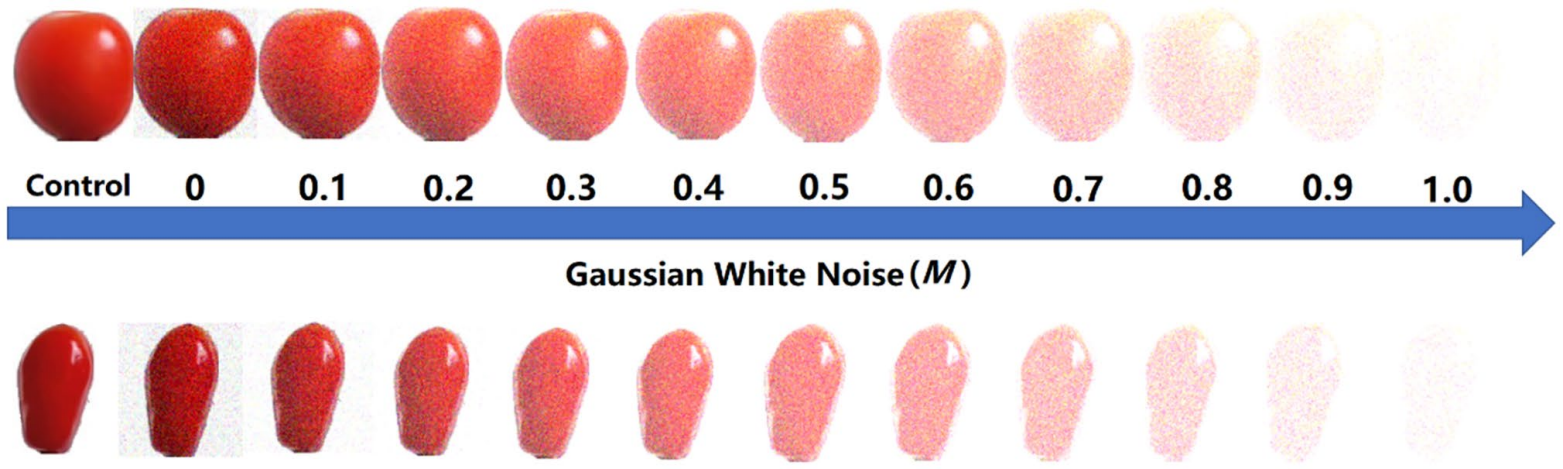
Tomato images with the addition of different levels of Gaussian white noise.

**Figure 2:**
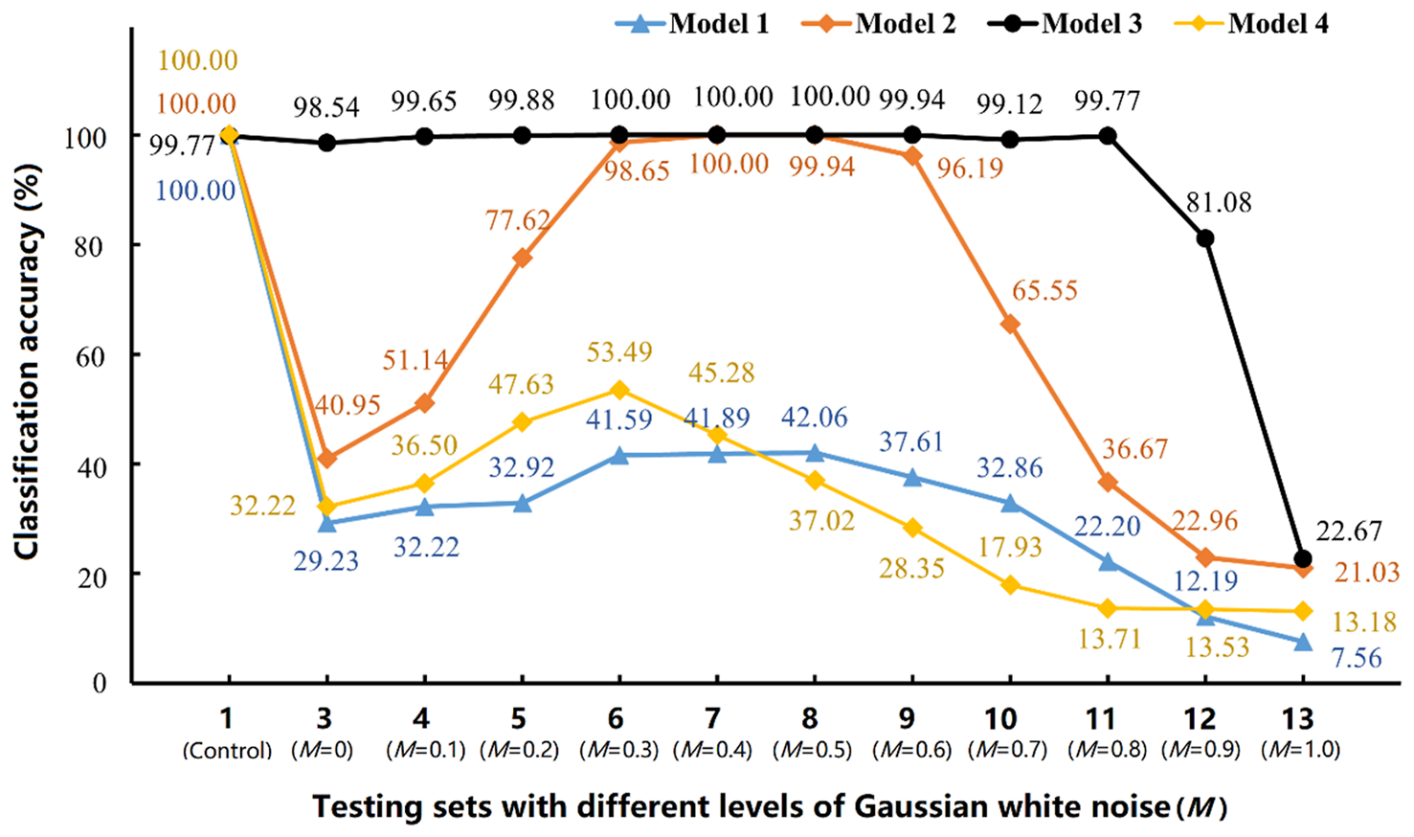
Performance variation of four DenseNet-201-based models (trained on different training sets) on twelve testing sets with different levels of noise.

The purpose of this part of the work was to simulate the poor quality images obtained in real scenarios by adding different levels of noise to tomato images^34^ and to find out the influence of image noise on the classification accuracy of a trained DenseNet-201-based model. Figure 1 shows the examples of tomato images with different levels of Gaussian white noise added, with *M* ranging from 0 to 1.0, and “Control” representing the original tomato image without the added noise. Obviously, the tomato images became increasingly unclear as the noise level increased, and when *M* > 0.7, they were difficult to recognize even with the human eye. Next, the trained DenseNet-201-based model (trained by training set 1) was tested on different testing sets to demonstrate the effect of image noise on the classification accuracy of the model. Specifically, as shown in Fig. 2 and Table 2, a total of twelve testing sets (1, 3–13) were used, where testing set 1 was the control set (the original testing set without added noise provided by Fruits-360 dataset), and testing sets 3–13 were based on testing set 1 with added different levels of Gaussian White noise (*M* from 0 to 1.0 in increments of 0.1), respectively. Model 1 in Fig. 2 represents the DenseNet-201-based model trained by training set 1 (in “Performance comparision of five CNN-based models” section). The curve of Model 1 shows that the classification accuracy drops sharply from 100.00 to 29.23% when the testing set starts to contain noise (*M* = 0), indicating that the trained model is sensitive to image noise^14^. Then, as the noise increases from *M* = 0.1 to *M* = 0.7, the classification accuracy fluctuates between 32.22 and 42.06%. And the classification accuracy continues to decrease as the noise level increases and is only 7.56% at *M* = 1.0 (testing set 13). This phenomenon indicates that although the trained Model 1 can achieve excellent performance on the testing sets of high-quality images, images containing noise significantly reduce its classification accuracy, which is unacceptable and limits the practical application of the model.

### Data augmentation of training sets

To address the above problem of Model 1, two data augmentation strategies, adding Gaussian white noise and geometric rotation, respectively, were adopted to generate additional training sets. Specifically, in Table 1, training set 3 is an augmented set consisting of two parts, one with all images of training set 1 and the other with Gaussian white noise (*M* = 0.5) added to each image of training set 1, thus making training set 3 twice the size of training set 1. Training set 4 consists of four parts, one of which is all the images of training set 1, and the other three parts are Gaussian white noise (*M* = 0.2, 0.5, and 0.8) added to each image of training set 1, respectively, so training set 4 is four times the size of training set 1. Meanwhile, training set 5 also consists of four parts, but is generated by geometric rotation, i.e., all the original images of training set 1, and the original images rotated by 90, 180, and 270 degrees respectively. Then, DesnseNet-201-based model was trained by training sets 3, 4 and 5 to obtain Models 2, 3 and 4, respectively, and the three models were tested on the twelve testing sets (1, 3–13) to examine their performance in classifying images containing different levels of noise.

As shown in Fig. 2, when the images in testing set begin to contain noise (*M* = 0), the performance of Models 2 and 4 is similar to that of Model 1, i.e., the classification accuracy decreases dramatically. After that, the classification accuracy of Model 2 starts to increase and reaches very high (over 96%) on testing set 6 (*M* = 0.3) to 9 (*M* = 0.6), but then decreases again when the noise *M* > 0.6. This trend is caused by two reasons: the size of training set 3 was two times larger than training set 1, so the overall performance of Model 2 was better than that of Model 1; Gaussian white noise (*M* = 0.5) was added to images of training set 3 resulted in Model 2 achieving high accuracy on the testing set 6 (*M* = 0.3) to 9 (*M* = 0.6), since the levels of image noise in these testing sets were the same or similar to those in training set 3. Inspired by the improved performance of Model 2, Model 3 was trained by training set 4, which was four times larger than training set 1. The performance of Model 3 is encouraging, as it not only overcomes the sensitivity of the model to image noise in the testing sets, but also maintains a high classification accuracy of about 99% on the ten testing sets (1, 3–11) with noise added (*M* ≤ 0.8). And the decrease in classification accuracy on testing sets 12 and 13 is due to the fact that the tomatoes in images were almost invisible after adding the noise (*M* = 0.9 and 1.0). Furthermore, Model 4 achieved very low classification accuracy on all testing sets, even lower than Model 1, indicating that the use of geometric rotation to augment the size of dataset did not have a positive impact on the improvement in model performance. This may be due to the fact that geometric rotation did not increase the diversity of the training data, whereas the addition of Gaussian white noise increased the diversity of the training data and thus allowed Model 3 to learn the underlying features used to distinguish between the different categories. Therefore, due to the poor performance of Model 4, we only compare the other three models in the next sections.

Models 1, 2 and 3 were similar in model size and classification time for a single image, at approximately 66 MB and 29 ms respectively. The training time of the three models increased with the increasing number of images in their training sets, i.e., 3743 s for Model 1, 6640 s for Model 2, and 16,180 s for Model 3. The relatively long training time for Model 3 is acceptable because firstly, in practice, the ultimate goal of a multiclass classification task is to achieve accurate classification of a single image with the shortest recognition time. Secondly, training the model is a one-off activity or at most a periodic training to maintain and update its performance, and finally it can be further shortened as computer hardware improved^35^. Based on the above results, it is shown that Model 3 has a stable performance and can accurately and quickly classify the images with different levels of noise, making it more suitable for practical agricultural applications.

### Feature visualization

**Figure 3:**
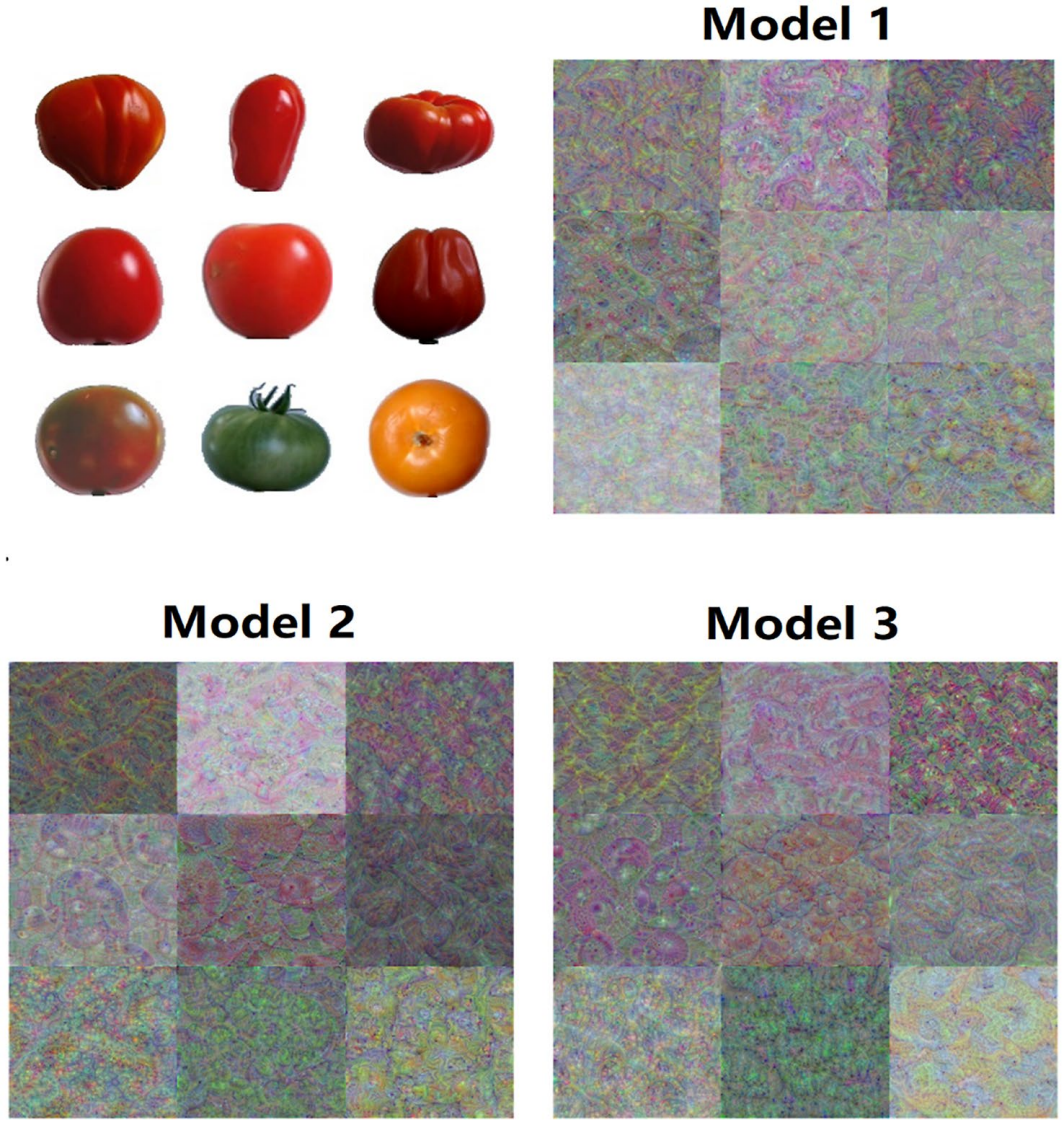
Sample images of nine types of tomatoes and feature visualization of the last fully connected layer of each trained model.

Since CNN can automatically learn features from raw image pixels during the training phase, feature visualization of a trained CNN is used to show its understanding of an image to humans^36,37^. Specifically, CNN generally builds understanding of an image in a hierarchical way over many layers, where earlier layers learn basic visual features such as edges or textures, while deeper layers can learn and integrate features learned by earlier layers into more abstract features such as patterns, parts, or objects^38^. Therefore, the feature visualization of the last fully connected layer of a trained model exhibits its learned common and high-level features of tomatoes in the training set, which are used to classify the different types of tomatoes in testing sets^39^. Figure 3 shows the sample images of each type of tomato in training set 1 and the corresponding feature visualization images generated by the three trained models. It can be seen that the feature visualization images generated by the different models for each class of tomatoes look similar but still differ in details, suggesting that image noise affects the learning and integration of features by the models. In addition, all the feature visualization images are colorful, complex, and abstract patterns that are difficult for us to describe and understand. This may be due to the fact that DenseNet-201 is very deep with 708 layers, and the presented feature visualization image was generated by layer 706 (the last fully connected layer). Although it was difficult for us to figure out what features they had learned, the truth is that Model 3 achieved excellent performance on complex testing sets containing noise based on its learned features.

### Strongest activations

**Figure 4:**
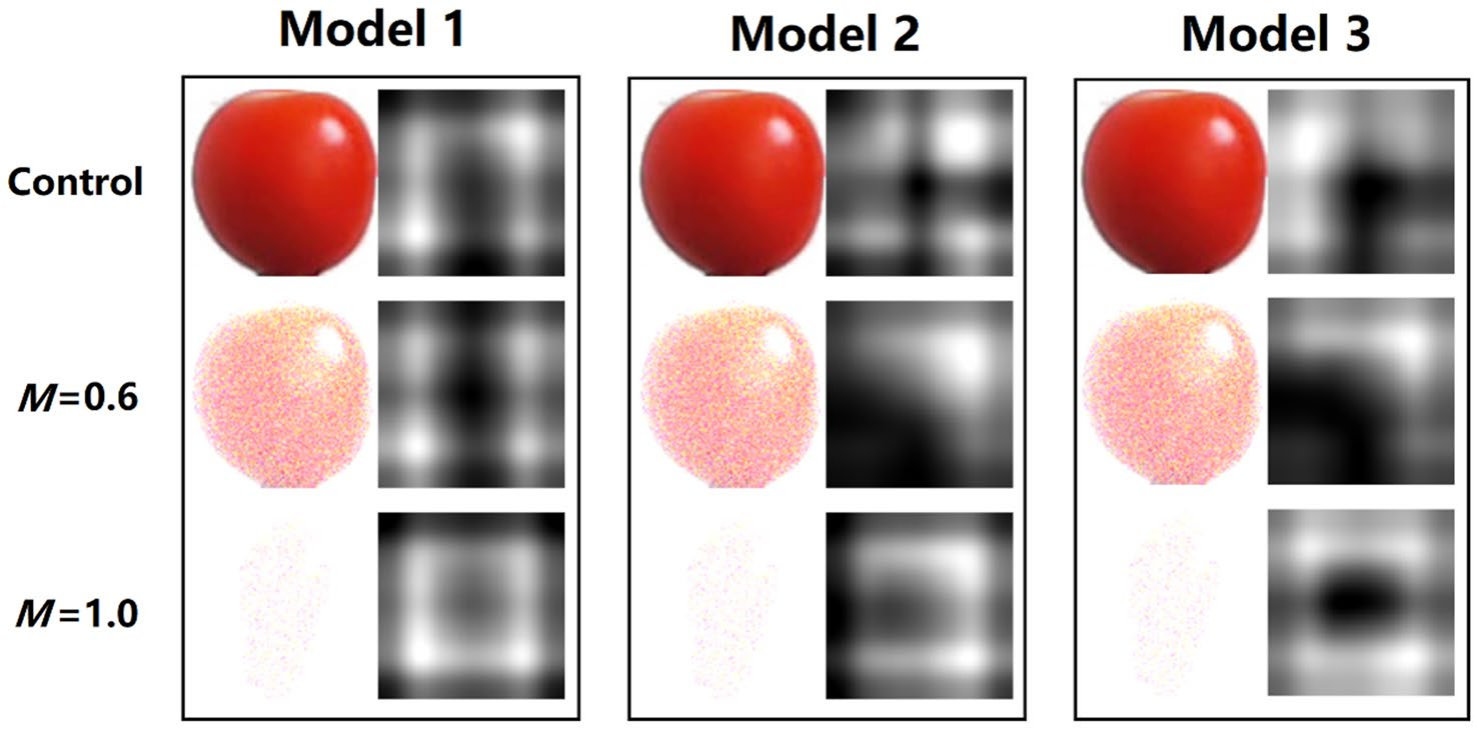
Randomly selected tomato images containing different levels of noise from testing sets 1, 9, and 13, and their corresponding strongest activations images generated by each trained model.

As Fig. 4 shows, one tomato image was randomly selected from testing sets 1 (Control), 9 (*M* = 0.6), and 13 (*M* = 1.0), respectively, and fed into each trained model to generate the corresponding strongest activations image. In the strongest activations images, the white pixels represent strong positive activation, which is the recognized areas by a trained model. Therefore, the aim of the work in this section was to show how a trained model recognizes a tomato and to demonstrate the differences in the recognition areas by different models for the same tomato image. First, for the tomato image in control set, the areas recognized by each model were almost identical and corresponded to all three models achieving about 100% classification accuracy on testing set 1 (Fig. 2), indicating that the areas they recognized should be correct. Second, for the tomato image with added noise (*M* = 0.6), the areas recognized by Models 2 and 3 were similar but significantly different from those recognized by Model 1, which corresponded to the high classification accuracy of 96.19% and 99.94% achieved by Models 2 and 3 respectively, on testing set 9, while the classification accuracy of Model 1 was only 37.61%. This phenomenon suggests that the areas recognized by Model 1 are incorrect and that the misidentified areas for tomatoes may be responsible for the low classification accuracy achieved by Model 1 on testing set 9. Last, for the tomato image with added noise (*M* = 1.0), since all three models had very low classification accuracy on testing set 13, their similar recognized areas of the tomato were probably incorrect. Thus, the above results indicate that the correct or incorrect target identification (strongest activations areas) by a trained model during the classification process will affect its final classification accuracy.

## Conclusion

In recently years, CNN-based methods have been successfully applied in agriculture and food. They are generally trained and tested on high quality image datasets and achieve high classification accuracy, as poor quality images can strongly interfere with target detection and classification of CNN, resulting in inaccurate output results. However, in practice, poor quality images are often obtained, which limits the application of CNN. Therefore, this study focuses on solving the problem of low accuracy of CNN in identifying and classifying poor quality images in real-world applications. We first used nine types of tomato images from the publicly available “Fruits-360” dataset to evaluate the performance of state-of-the-art CNNs and chose DenseNet-201 with transfer learning as the optimal model. Twelve testing sets were then constructed by adding vary levels of Gaussian white noise in order to mimic the poor-quality images obtained in practice. The poor performance of Model 1 on the twelve testing sets showed that DenseNet-201-based model was very sensitive to image noise. Next, training DenseNet-201-based models with the augmented training sets obtained by adding Gaussian white noise to the images solved this problem, as it not only expanded the size of training sets but also increased the diversity of the training data. Furthermore, the feature visualization and strongest activations of the three trained DenseNet-201-based models were investigated to compare the differences between the different models, and the visual evidence obtained can be used to gain insight into the internal structure or working principle of deep network as a “black box”. In conclusion, the results showed that Model 3 achieved superior performance in identifying and classifying both high and poor-quality images, and thus can be used as a generic intelligent tomato classification system in practical applications.

## Data Availability

The datasets generated during the current study are available from the corresponding author on reasonable request.
