# Author Correction: A generic intelligent tomato classification system for practical applications using DenseNet-201 with transfer learning

**DOI:** 10.1038/s41598-021-98942-5

**Published:** 2021-09-21

**Authors:** Tao Lu, Baokun Han, Lipin Chen, Fanqianhui Yu, Changhu Xue

**Affiliations:** 1 College of Mechanical and Electronic Engineering, Shandong University of Science and Technology, Qingdao, 266590 China; 2 School of Mechanical and Automotive Engineering, Qingdao University of Technology, Qingdao, 266520 China; 3 College of Food Science and Engineering, Ocean University of China, Qingdao, 266003 China; 4 Laboratory of Marine Drugs and Biological Products, Pilot National Laboratory for Marine Science and Technology (Qingdao), Qingdao, 266237 China

Correction to: *Scientific Reports* https://doi.org/10.1038/s41598-021-95218-w, published online 04 August 2021

In the original version of this Article, Affiliation 1 and 2 were not listed in the correct order. The correct affiliations are listed below:

Affiliation 1:

College of Mechanical and Electronic Engineering, Shandong University of Science and Technology, Qingdao, 266590, China

Affiliation 2:

School of Mechanical and Automotive Engineering, Qingdao University of Technology, Qingdao, 266520, China

The original Article has been corrected.

